# Supplementary material for: Gene Expression Analysis of Aggressive Clinical T1 Stage Clear Cell Renal Cell Carcinoma for Identifying Potential Diagnostic and Prognostic Biomarkers
Source: Cancers (Basel). 2020 Jan 16;12(1):222. doi: 10.3390/cancers12010222 (PMC7017065; doi:10.3390/cancers12010222)
Supplement: Supplementary file 1 [file cancers-12-00222-s001.zip › cancers-689725-supplementary materials.pdf]

cancers

Supplementary Materials

Gene Expression Analysis of Aggressive Clinical T1 Stage Clear Cell Renal Cell Carcinoma for Identifying Potential Diagnostic and Prognostic Biomarkers

Jee Soo Park, Phillip M. Pierorazio, Ji Hyun Lee, Hyo Jung Lee, Young Soun Lim, Won Sik Jang, Jongchan Kim, Seung Hwan Lee, Koon Ho Rha, Nam Hoon Cho and Won Sik Ham *

Table S1. PCR primer sequences of target genes.

	Primer Sequences	
	**Sense**	**Antisense**
**FOXC2**	5′-GAT CAC CTT GAA CGG CAT CT-3′	5′-ACC TTG ACG AAG CAC TCG TT-3′
**CLIP4**	5′-GCA TCA TGC CAG GAA ATT CT-3′	5′-TTT GTT GGA CCT GAG GAA CC-3′
**PBRM1**	5′-TGA TGG CCA ACA AGT ACC AA-3′	5′-AGA TCA AAG ACT CCG GCT CA-3′
**SETD2**	5′-TCA CAA GGC AGA CTC AGT GG -3′	5′-CTG CTG TCT TGG GCT TTT TC-3′
**BAP1**	5′-GCC TGA GGA GTC CAA GTC AG-3′	5′-CTG GAG GCT TCA CCA CTA GC-3′
**KDM5C**	5′-GTC ATT TGC AAC CCC TGA GT-3′	5′-AAT GGG ATG AGG GGT AAA GG-3′
**AQP1**	5′-CAA CTT CAG CAA CCA CTG GA-3′	5′-GTC GGC ATC CAG GTC ATA CT-3′
**DDX11**	5′-TCT CTT GGC TCC GTG ACT TT-3′	5′-TTT AGT CGG TCC ACC AGG TC-3′
**BAIAP2L1**	5′-GGC AGG AGA CCT GTG TTG AT-3′	5′-AGC CTG AGG AGT TCC AGA CA-3′
**GAPDH**	5′-CAG CCT CAA GAT CAT CAG CA-3′	5′-GGT GCT AAG CAG TTG GTG GT-3′

Table S2. Patient baseline characteristics.

	Clinical T1 Stage ccRCC (n = 24)	RCC with Aggressive Characteristics (n = 12)	RCC without Aggressive Characteristics (n = 12)	*P* Value[a]
**Gender, n (%)**				
**Male**	23 (95.8%)	12 (100.0%)	11 (91.7%)	1.000
**Female**	1 (4.2%)	0 (0.0%)	1 (8.3%)	
**Age (years)**	66.0 ± 8.8	63.6 ± 9.1	68.4 ± 8.2	0.185
**BMI (kg/m^2)**	25.1 ± 3.9	26.4 ± 4.7	23.7 ± 2.3	0.096
**Radical surgery, n (%)**	15 (62.5%)	8 (66.7%)	7 (58.3%)	1.000
**Tumor size (cm)**	4.9 ± 1.6	4.9 ± 1.6	4.9 ± 1.6	0.960
**Fuhrman grade, n (%)**				
**1**	0 (0.0%)	0 (0.0%)	0 (0.0%)	0.335
**2**	9 (37.5%)	4 (33.3%)	5 (41.7%)	
**3**	13 (54.2%)	6 (50.0%)	7 (58.3%)	
**4**	2 (8.3%)	2 (16.7%)	0 (0.0%)	
**3-4, n (%), vs. 1-2**	15 (62.5%)	8 (66.7%)	7 (58.3%)	1.000
**Invasion (perinephric/sinus fat/vascular), n (%)**	15 (62.5%)	7 (58.3%)	8 (66.7%)	1.000
**Positive nodal status, n (%)**	1 (4.2%)	1 (8.3%)	0 (0.0%)	1.000
**Synchronous metastasis, n (%)**	6 (25.0%)	6 (50.0%)	0 (0.0%)	0.014*
**Metastatic sites, n (%)**				
**Lung, n (%)**	4 (57.1%)	4 (57.1%)		
**Bone, n (%)**	3 (42.9%)	3 (42.9%)		
**Other, n (%)**	0 (0.0%)	0 (0.0%)		
**Recurrences, n (%)**	7 (29.2%)	7 58.3%)	0 (0.0%)	0.005*
**Time to recur (months)**	25.3 ± 17.5	25.3 ± 17.5		
**Recur sites**				
**Single, n (%)**	2 (28.6%)			
**Multiple, n (%)**	5 (71.4%)			
**Local, n (%)**	2 (20.0%)			
**Liver, n (%)**	3 (30.0%)			
**Bone, n (%)**	3 (30.0%)			
**Lymph node, n (%)**	2 (20.0%)			
**Other, n (%)**	0 (0.0%)			
**Cancer-specific death, n (%)**	8 (33.3%)	8 (66.7%)	0 (0.0%)	0.001*
**Survival time[b] (months)**	44.1 ± 21.0	39.3 ± 20.3	48.9 ± 21.5	0.273

Data are shown as mean ± SD or number of subjects (%); BMI, body mass index; ccRCC, clear cell renal cell carcinoma; RCC, renal cell carcinoma; [a] *p* values were calculated using the t-test for continuous variables and the chi-square test or Fisher's exact test for categorical variable; Star (*) indicates $p < 0.05$; [b] Survival time was defined as the time from nephrectomy until the patient's death or the last time that the patient was known to be alive.

Table S3. Mutational frequencies of the candidate aggressiveness-associated genes.

Gene Symbol	Gene Title	Mutation Frequency			*P*- value[a]
		Clinical T1 stage ccRCC (n = 24)	RCC with aggressive characteristics (n = 12)	RCC without aggressive characteristics (n = 12)	
PBRM1	Polybromo 1	11/24 (45.8%)	6/12 (50.0%)	5/12 (41.7%)	0.682
BAP1	BRCA1 associated protein-1	6/24 (25.0%)	4/12 (33.3%)	2/12 (16.7%)	0.640
SETD2	SET domain-containing 2	24/24 (100.0%)	12/12 (100.0%)	12/12 (100.0%)	-
KDM5C	Lysine-specific demethylase 5C	9/24 (37.5%)	6/12 (50.0%)	3/12 (25.0%)	0.400
FOXC2	Forkhead box protein C2	6/24 (25.0%)	4/12 (33.3%)	2/12 (16.7%)	0.640
CLIP4	Cytoskeleton-associated protein-glycine rich (CAP-Gly) domain-containing linker protein family member 4	8/24 (33.3%)	6/12 (50.0%)	2/12 (16.7%)	0.193

RCC, renal cell carcinoma; [a] *p* -values were calculated using the chi-square test.

Table S4. Expression of combination of DDX11, TMEM38B, and PRUNE2 by oncological outcomes (cancer-specific death and recurrence) (*in addition to Table 4*).

	**Cancer-specific death**				
	RCC with cancer-specific death (n = 8)	RCC without cancer-specific death (n = 16)	*p*-value[a]	Multivariate OR (95% CI)	*p*-value[b]
**DDX11+&TMEM38B−&PRUNE2−**	6/8 (75.0%)	1/16 (6.2%)	0.001	45.000 (3.408-594.116)	0.004
	**Recurrence**				
	RCC with recurrence (n = 7)	RCC without recurrence (n = 17)	*p*-value[a]	Multivariate OR (95% CI)	*p*-value[b]
**DDX11+&TMEM38B−&PRUNE2−**	6/7 (85.7%)	1/17 (5.9%)	<0.001	96.000 (5.145–1791.219)	0.002

Data are shown as the number of patients (%); [a] *p*-value calculated using the chi-square test or Fisher's exact test; [b] *p* -value calculated using logistic regression for multivariate analysis; DDX11+, FPKM (fragments per kilobase of exon per million fragments mapped) >20.0; TMEM38B−, FPKM < 5.0; PRUNE2−, FPKM < 32.0.

Table S5. Comparison of expression levels of target genes according to Fuhrman grade in clear cell renal cell carcinoma (≤4 cm).

	Fuhrman Grade				
	**Low grade (Grade 1 + 2) (n = 37)**	**High grade (Grade 3 + 4) (n = 33)**	***p*-value[a]**	**Multivariate OR (95% CI)**	***p*-value[b]**
**Gender, n (%)**					
**Male**	32 (86.5%)	27 (81.8%)	0.592		
**Female**	5 (13.5%)	6 (18.2%)			
**Age (yrs)**	51.78 ± 14.09	55.70 ± 12.76	0.229		
**Tumor size (cm)**	2.03 ± 0.77	2.44 ± 0.86	0.039	2.005 (1.060–3.795)	0.033
**Gene expression levels**					
**FOXC2**	0.3947 ± 0.9919	0.0647 ± 0.0749	0.051		
**CLIP4**	0.1653 ± 0.2514	0.1269 ± 0.1562	0.451		
**PBRM1**	0.1130 ± 0.1462	0.0821 ± 0.1038	0.317		
**SETD2**	0.0107 ± 0.0298	0.0073 ± 0.0140	0.544		
**BAP1**	0.2714 ± 0.7583	0.0230 ± 0.0525	0.054		
**KDM5C**	0.0226 ± 0.0458	0.0163 ± 0.0304	0.509		
**AQP1**	0.0037 ± 0.0057	0.0046 ± 0.0071	0.582		
**DDX11**	0.0059 ± 0.0057	0.0148 ± 0.0215	0.027	1.140×10^{24} ($1.556–8.314 \times 10^{47}$)	0.048
**BAIAP2L1**	0.0288 ± 0.0393	0.0208 ± 0.0259	0.325		

Data are shown as mean±SD or number of subjects (%); [a] *p*-value calculated using t-test for continuous variables and chi-square test for categorical variables; [b] *p* -value calculated using logistic regression for multivariate analysis.

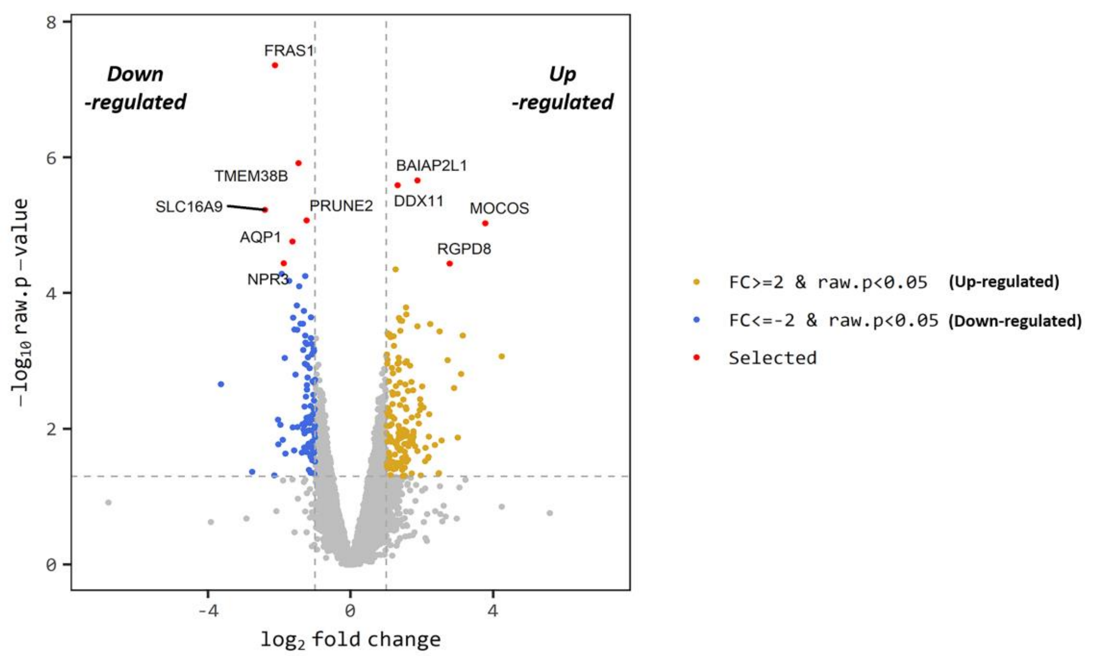

Figure S1. Volcano plots showing a comparison of gene expression levels in aggressive ccRCC and non-aggressive ccRCC. Yellow: $\log_2$ fold change (FC) ≥ 2, $p < 0.05$; blue: $\log_2$ FC ≤ 2, $p < 0.05$; red: selected genes.

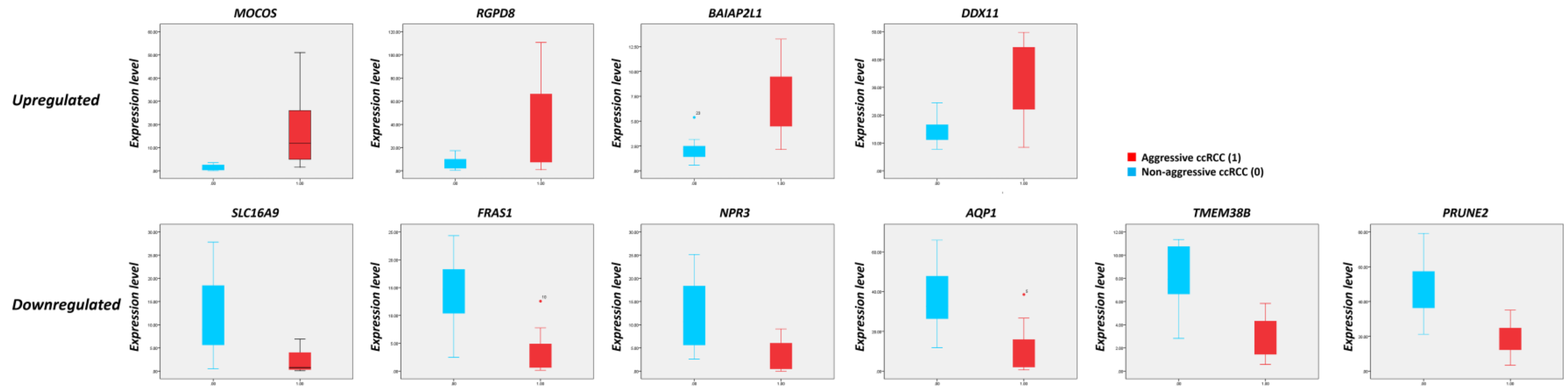

Figure S2. Box plots showing a comparison of expression levels of 10 selected genes in aggressive and non-aggressive ccRCC.

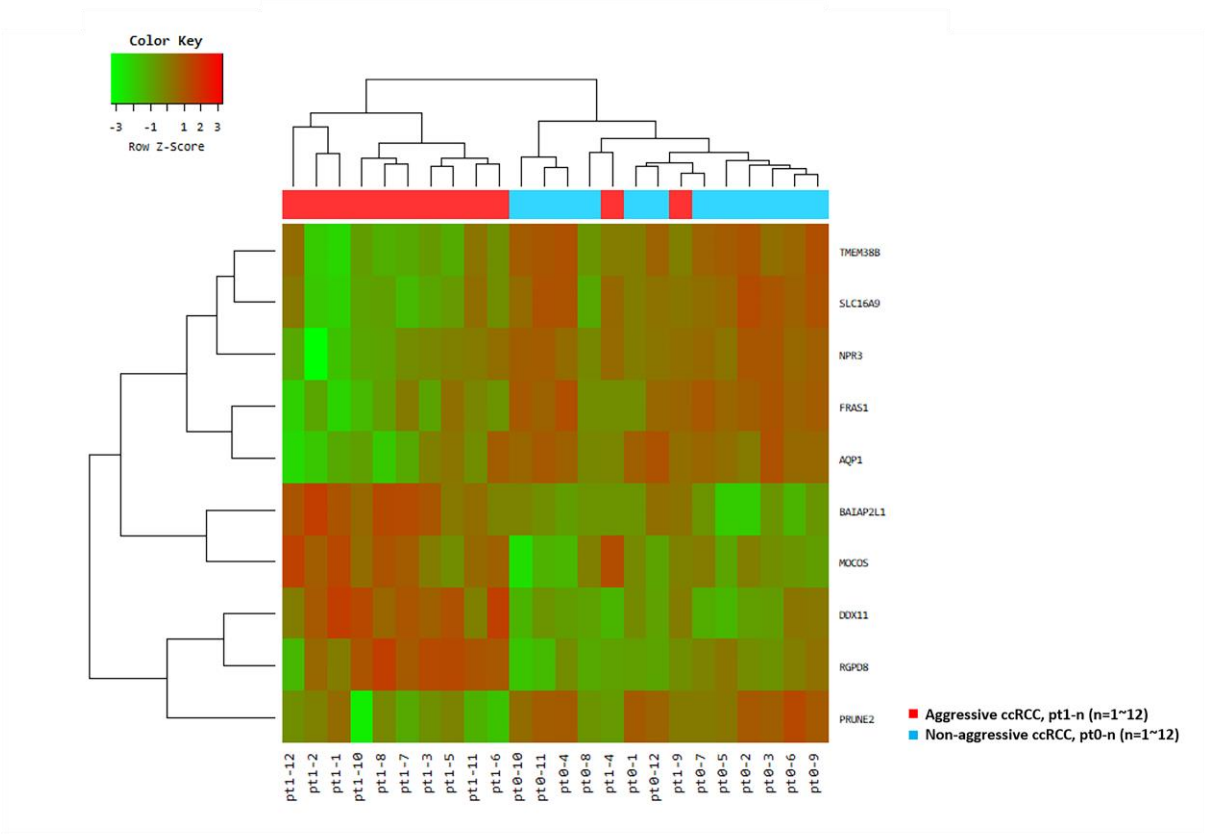

Figure S3. Supervised hierarchical clustering analysis (red, high relative expression; green, low relative expression) of aggressive ccRCC patients (n = 12, red) versus matched non-aggressive ccRCC patients (n = 12, blue) based on the 10 selected genes.

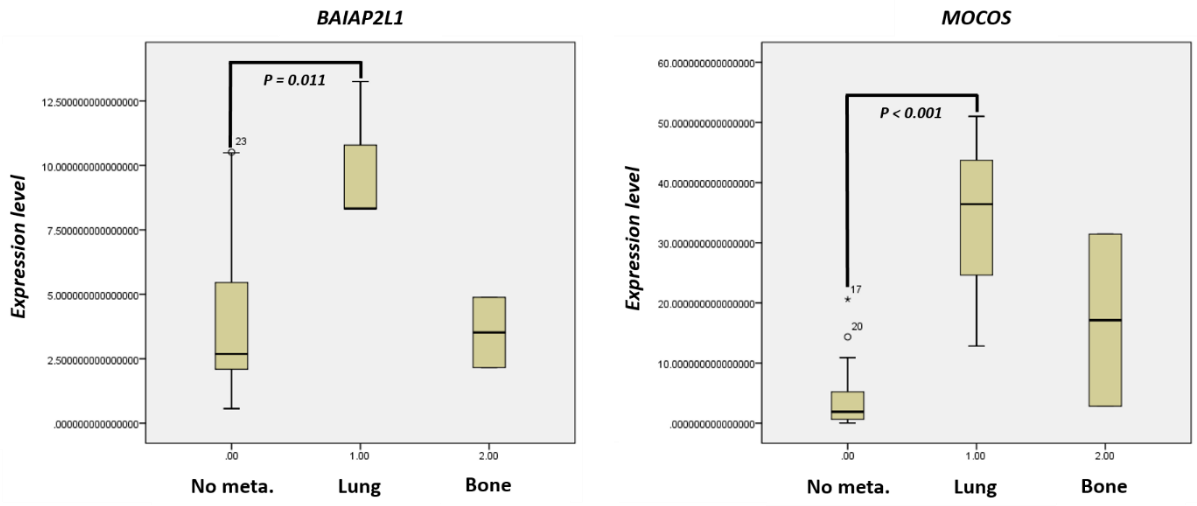

Figure S4. Expression levels of *BAIAP2L1* and *MOCOS* according to the synchronous metastatic sites (lung and bone) shown as box plots. The box depicts the 25% and 75% quartile borders; horizontal bar and median value.

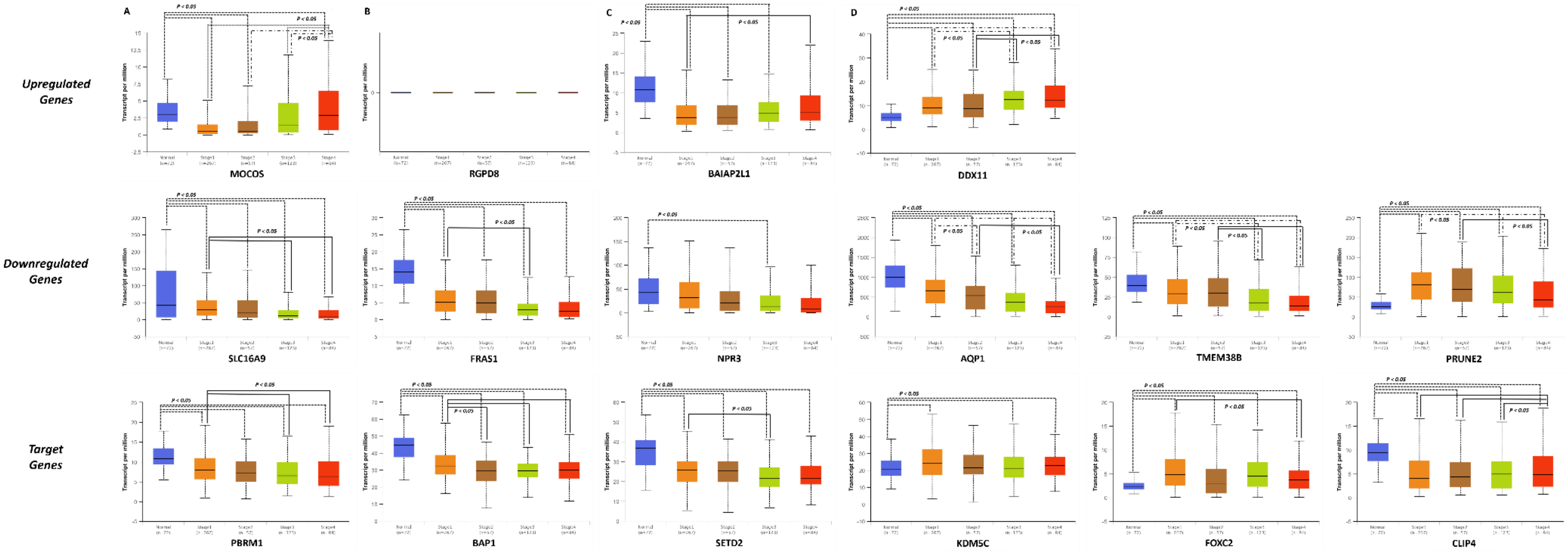

Figure S5. Validation of the expression of 16 genes in the TCGA database according to stage.

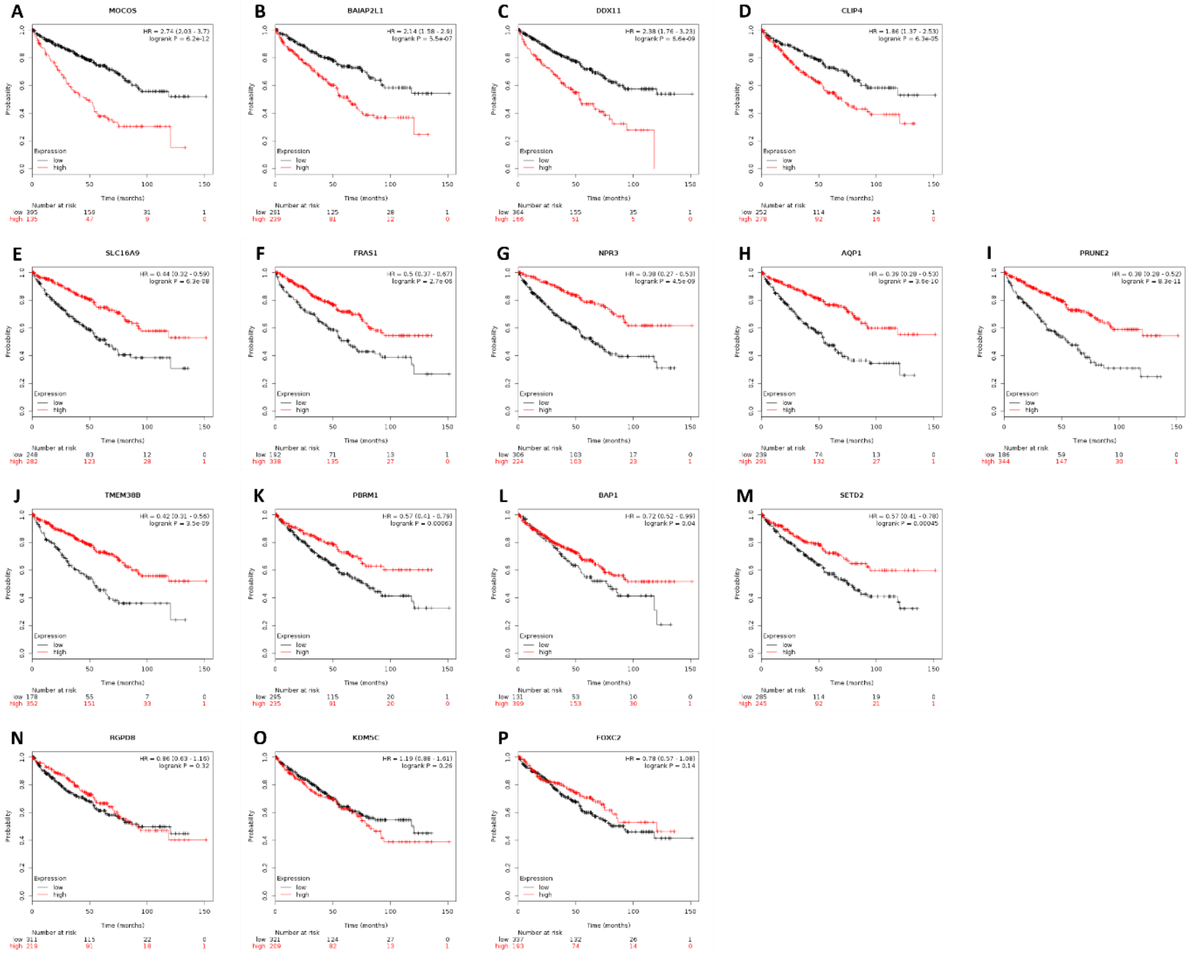

Figure S6. Association of the 16 genes with overall survival (OS) of clear cell renal cell carcinoma (ccRCC) patients, as analyzed by Kaplan-Meier survival plots. (**A–D**) High expression of *MOCOS*, *BAIAP2L1*, *DDX11*, and *CLIP4* was associated with poor OS of ccRCC patients. (E-M) Low expression of *SLC16A9*, *FRAS1*, *NPR3*, *AQP1*, *PRUNE2*, *TMEM38B*, *PBRM1*, *BAP1*, and *SETD2* was associated with poor OS of ccRCC patients. (**N–P**) Expression level of *RGPD8*, *KDM5C*, and *FOXC2* was not related.

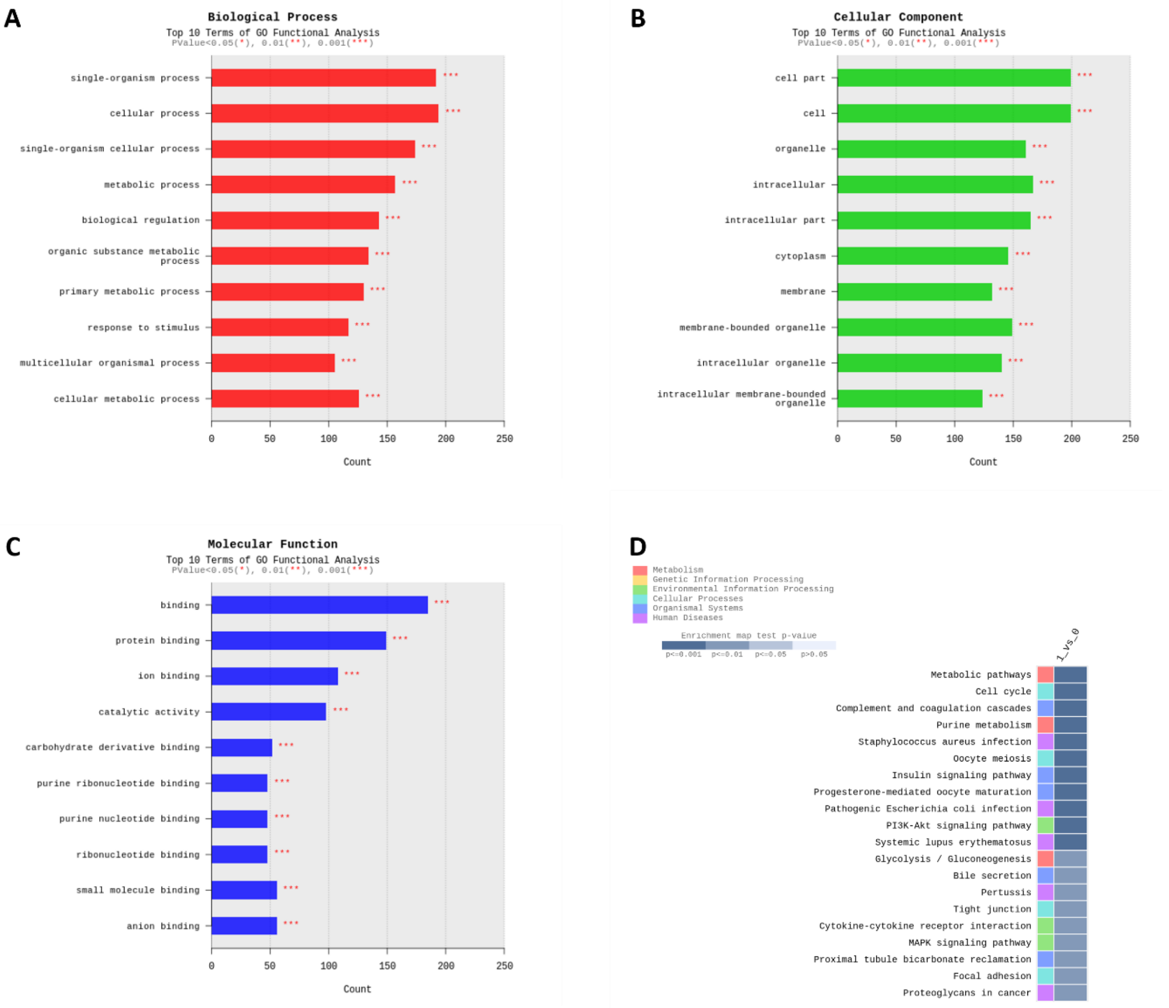

Figure S7. GO annotation and pathway enrichment analysis. Top 10 terms in (**A**) BP (biologic process) category, (**B**) CC (cellular component) category, and (**C**) (molecular function) category. (**D**) Significantly associated KEGG pathways.

Supplementary – Methods

Supplementary Method 1: Patients and tissues

Details of exclusion criteria and data on clinicopathologic features

Exclusion criteria

Patients who had received neoadjuvant or adjuvant systemic therapy, or who had a history of inherited von Hippel-Lindau disease or synchronous/metachronous bilateral RCC, were excluded. Patients with very little or no tumor tissue (less than 5% of the area occupied by invasive cancer cells), or with insufficient RNA or RNA of inadequate quality, were also excluded. In addition, cases recurring within six months after surgery in the absence of adequate imaging were excluded to eliminate the possibility of undetected metastasis.

Data on clinicopathologic features

Data on clinicopathologic features, including age, sex, body mass index (BMI), tumor size, presence of metastasis, metastatic sites, recurrence, recurrence sites, time to recurrence, cancer-specific death, survival time (defined as the time from nephrectomy until the patient's death, or the last time that the patient was known to be alive), positive nodal status, and invasion status were recorded for each

patient. Histological subtype was assessed according to the 2004 WHO Renal Neoplasms guidelines,[1] and Fuhrman grade, invasion (perinephric/sinus fat or microscopic vascular invasion), and lymph node involvement were assessed according to the 2010 American Joint Committee on Cancer system.[2] Grading was based on a standardized four-tier system.[3]

Supplementary Method 1 – References:

1. Eble, J.N.; Sauter, G.; Epstein, J.; Sesterhenn, I. World Health Organization classification of tumors. Pathology and genetics of tumours of the urinary system and male genital organs. Lyon: IARC Press, 2004.
2. Edge, S.B.; Compton, C.C. The American Joint Committee on Cancer: the 7th edition of the AJCC cancer staging manual and the future of TNM. Ann Surg Oncol 2010, 17, 1471.
3. Fuhrman, S.A.; Lasky, L.C.; Limas, C. Prognostic significance of morphologic parameters in renal cell carcinoma. Am J Surg Pathol 1982, 6, 655.

Supplementary Method 2: RNA extraction and sequencing

Details of RNA extraction and sequencing methodology

RNA extraction

Total RNA concentration was determined using a Quant-iT PicoGreen double-stranded (ds) DNA assay Kit (Invitrogen, Carlsbad, CA, USA). To determine fragment sizes (DV200 metrics, % of RNA fragments > 200 bp), samples were run on a TapeStation 4200 RNA screentape (Agilent Technologies, Palo Alto, CA, USA).

Sequencing methodology

Total RNA was first fragmented into small pieces using divalent cations under elevated temperature. The cleaved RNA fragments were copied onto the first strand complementary DNA (cDNA) using the Superscript II Reverse Transcriptase Kit (Invitrogen, Cat #18064014) and random primers. This step was followed by second strand cDNA synthesis using DNA Polymerase I, RNase H, and dUTP.

After second strand cDNA synthesis, cDNA fragments underwent an end repair process with the addition of a single 'A' base, followed by ligation of the adapters. The products were then purified and enriched by PCR to create the cDNA library. All libraries were normalized, and four libraries were pooled into a single hybridization/capture reaction. Pooled libraries were incubated with a cocktail of biotinylated oligos corresponding to the coding regions of the genome. Targeted library molecules were captured via hybridized biotinylated oligo probes using streptavidin-conjugated beads. After two rounds of hybridization/capture reactions, the enriched library molecules were subjected to a second round of PCR amplification.

Supplementary Method 3: RNA sequencing analysis

Details of RNA sequencing analysis and principal component analysis

RNA sequencing analysis

Paired end sequencing reads of cDNA libraries (101 bp) were generated with the NovaSeq6000 instrument (Illumina), and the quality of sequencing data was assessed with FastQC (version 0.10.0; http://www.bioinformatics.babraham.ac.uk/projects/fastqc). For data preprocessing, low quality bases and adapter sequences in reads were trimmed using Trimmomatic (version 0.32).[1] Trimmed reads were aligned to the reference human genome ([University of California Santa Cruz] UCSC hg19) using HISAT2 (version 2.1.0)[2], a splice-aware aligner. Transcript assembly of known transcripts, novel transcripts, and alternative splicing transcripts was processed by StringTie (version 1.3.4).[3,4]

Read count data of filtered genes were normalized by RLE (Relative Log Expression) normalization with DESeq2. The nbinomTest function in DESeq2 was used to test for differential expression, with the output set to display fold change, *P*-value, and false discovery rate (FDR) values. FDR was corrected by adjusting the *P*-values using the Benjamini-Hochberg algorithm. DEGs were determined by |fold change| ≥ 2 and *P*-value < 0.05.

Principal component analysis

Principal components (PCs) were uncorrelated and ordered such that the kth PC had the kth largest variance among all PCs. The first few PCs usually contain most of the data variations; however, the first two PCs of these data had fewer variations than the other PCs. PCs 1 and 2 were generated. The statistical significance of fold change in expression was determined using paired t-tests, with the null hypothesis such that no difference existed between the two values. A volcano plot (X-axis: $\log_2$ fold change, Y-axis: $-\log_{10}$ *P*-value) was drawn to identify genes with large fold changes that were also statistically significant.

Supplementary Method 3 – References:

4. Bolger, A.M.; Lohse, M.; Usadel, B. Trimmomatic: a flexible trimmer for Illumina sequence data. Bioinformatics. 2014, 30, 2114.
5. Kim D, Langmead B, Salzberg SL. HISAT: a fast-spliced aligner with low memory requirements. Nat Methods. 2015, 12, 357.
6. Pertea, M.; Pertea, G.M.; Antonescu, C.M.; Chang, T.C.; Mendell. J.T.; Salzberg, S.L. StringTie enables improved reconstruction of a transcriptome from RNA-seq reads. Nat Biotechnol. 2015, 33, 290.
7. Pertea, M.; Kim, D.; Pertea, G.M.; Leek, J.T.; Salzberg, S.L. Transcript-level expression analysis of RNA-seq experiments with HISAT, StringTie and Ballgown. Nat Protoc. 2016, 11, 1650.

Supplementary Method 4: Variant calling

Details of sequencing methodology and annotation of variants

For variant calling of RNA-seq data, trimmed reads were aligned to the human genome (UCSC hg19) with the Spliced Transcripts Alignment to Reference (STAR) aligner, and duplications were marked and discarded using Picard MarkDuplicates (http://picard.sourceforge.net/). Next, aligned reads used in analysis were created through Split 'N' Trim, mapping quality reassignment, indel realignment, and base recalibration process. Alignment reads used for variant calling were created using Genome Analysis Toolkit (GATK) - HaplotypeCaller followed by standard hard variant filtering for each sample based on Fisher Strand values (FS > 30.0) and Qual By Depth values (QD < 2.0) in the VariantFiltration module of GATK.[27,28] For our target gene list (*PBRM1*, *BAP1*, *SETD2*, *KDM5C*, *FOXC2*, and *CLIP4*), the frequency of alterations in all samples is represented on the heatmap. Individual genes are represented as rows, and individual patients are represented as columns.

Supplementary Method 4 – References:

8. Van der Auwera, G.A.; Carneiro, M.O.; Hartl, C.; Poplin, R.; Del Angel, G.; Levy-Moonshine, A.; Jordan, T.; Shakir, K.; Roazen, D.; Thibault, J.; Banks, E.; Garimella, K.V.; Altshuler, D.; Gabriel, S.; DePristo, M.A. From FastQ data to high confidence variant calls: The Genome Analysis Toolkit best practices pipeline. *Curr Protoc Bioinformatics.* **2013**, *43*, 1.
9. Chandrashekar, D.S.; Bashel, B.; Balasubramanya, S.A.H.; Creighton, C.J. Ponce-Rodriguez, I.; Chakravarthi, B.V.S.K.; Varambally, S. UALCAN: A portal for facilitating tumor subgroup gene expression and survival analyses. *Neoplasia.* **2018**, *19*, 649.

Supplementary Method 5: qRT-PCR

Details of sequencing methodology

qRT-PCR was performed with a Power SYBR® Green Master Mix (Thermo Fisher, Cat No. A25742, USA) in a 10-μL reaction volume comprising 5 μL of SYBR® Green master PCR mix, 1 μL of each forward and reverse primer (10 pmol), 1 μL of diluted cDNA template, and sterile distilled water. Conditions for the amplification of genes were as follows: initial denaturation at 95 °C for 10 min; 40 cycles of denaturation at 95 °C for 15 s, annealing at 58 °C for 60 s, and elongation at 72 °C for 60 s; final elongation was performed at 72 °C for 5 min. qRT-PCR was performed on the ABI StepOnePlus Real-Time PCR System (Applied Biosystems, Foster City, CA, USA). All quantifications were performed with GAPDH as a reference gene for standardization of relative expression levels. PCR primer sequences are presented in Supplementary Table 1 (appendix pp 1). Relative gene expression was analyzed using the $2^{-\Delta\Delta Ct}$ method and the results are expressed as the percent change with respect to control values. At least three replicates of qRT-PCR experiments were performed and analyzed by a blinded investigator.

Supplementary Method 6: GO and KEGG analysis

Details of GO and KEGG analysis

GO analysis

Each GO bar plot represents the top 10 associated GO gene set terms of each GO category filtered by a modified Fisher's exact test, with a *P*-value less than 0.05 considered to indicate statistical significance.

KEGG analysis

The sorted heatmap of KEGG enrichment represents pathways significantly enriched, filtered by a *P*-value less than 0.05, as determined by Fisher's exact test, for determination of statistical significance of each gene set pathway.

Supplementary Method 7: Validation of gene expression, survival analyses, and statistical analyses

Details of UALCAN and Kaplan-Meier plotter database analysis

UALCAN

UALCAN (http://ualcan.path.uab.edu), an online tool used to analyze gene expression data from The Cancer Genome Atlas (TCGA) database (including 72 normal kidney tissues and 533 primary tumors), was used to validate the expression of 10 newly identified genes and six target genes according to cancer stage. A *P*-value less than 0.05 was considered to indicate a statistically significant difference.[1]

Kaplan-Meier plotter database

The Kaplan-Meier plotter database (http://kmplot.com) is an online tool that contains data on the relationships between 54,675 genes and prognosis of patients across 10,461 cancer samples, and is used to evaluate the prognostic values of genes in cancer patients. This platform was applied to analyze the associations between 16 genes (10 newly identified and 6 target genes) and overall patient survival.[2] Differences in survival rates were statistically analyzed between patients with high and low expression levels of 16 genes. Patients were categorized into low or high groups by computing all possible cutoff values between the lower and upper quartiles, and the best performing threshold was used as a cutoff. The hazard ratio (HR) and 95% confidence intervals (CIs) were calculated. A *P*-value less than 0.05 was considered to indicate a statistically significant difference.

Statistical analyses

Results are reported as the mean ± standard deviation for continuous variables and as a percentage for categorical variables. For the univariate analysis, a *t*-test was used to compare continuous variables, and a Chi-square test or Fisher's exact test was used to compare categorical variables. Multivariate analysis was based on logistic regression. Frequency of candidate genes suspected to be associated with cancer aggressiveness in patients with aggressive ccRCC was compared to that in patients with non-aggressive ccRCC. *DDX11* positive tumors were categorized as the FPKM values higher than 20.0. Tumors were categorized as *TMEM38B* negative and *PRUNE2* negative. The FPKM values were less than 5.0 and 32.0, respectively. Cancer-specific survival (CSS) and recurrence-free survival (RFS) were assessed using Kaplan-Meier estimates and compared using the log-rank test. Hazard ratios (HRs) were obtained from Cox regression analyses after adjusting for patient age. For the PCR analysis from frozen tissue, a t-test and Chi-square test were used to compare continuous variables and categorical variables, respectively. Multivariate analysis was based on logistic regression, including all risk factors that were significantly associated in univariate analysis. SPSS software version 23.0 (IBM Corp., Armonk, NY) was used for all statistical analyses. All statistical tests were two-tailed, and a *P*-value less than 0.05 was considered statistically significant.

Supplementary Method 7 – References:

10. Lanczky. A.; Nagy. A.; Bottai, G.; Munkácsy, G.; Szabó, A.; Santarpia, L.; Győrffy, B. miRpower: A web-tool to validate survival-associated miRNAs utilizing expression data from 2178 breast cancer patients. Breast Cancer Res Treat. 2016, 160, 439.
11. DePristo, M.A.; Banks, E.; Poplin, R.; Garimella, K.V.; Maguire, J.R.; Hartl, C.; Philippakis, A.A.; del Angel, G.; Rivas, M.A.; Hanna, M.; McKenna, A.; Fennell, T.J.; Kernytsky, A.M.; Sivachenko, A.Y.; Cibulskis, K.; Gabriel, S.B.; Altshuler, D.; Daly, M.J. A framework for variation discovery and genotyping using next-generation DNA sequencing data. Nat Genet. 2011, 43, 491.
